# Nipple Hibernoma in a Dog: A Case Report With Literature Review

**DOI:** 10.3389/fvets.2021.627288

**Published:** 2021-05-12

**Authors:** Irina Amorim, Fatima Faria, Marian Taulescu, Cristina Taulescu, Fatima Gärtner

**Affiliations:** ^1^Department of Pathology and Molecular Immunology of the Institute of Biomedical Sciences Abel Salazar, University of Porto, Porto, Portugal; ^2^Instituto de Investigação e Inovação em Saúde, Universidade do Porto, Porto, Portugal; ^3^Institute of Molecular Pathology and Immunology of the University of Porto, Porto, Portugal; ^4^Department of Pathology, Faculty of Veterinary Medicine, University of Agricultural Sciences and Veterinary Medicine Cluj-Napoca, Cluj-Napoca, Romania; ^5^Synevovet Laboratory, Bucharest, Romania; ^6^Visionvet Veterinary Ophthalmology Clinic, Bologna, Italy

**Keywords:** brown adipose tissue, hibernoma, UCP1, cancer, canine

## Abstract

This report provides a clinical, histological, and immunohistochemical description of an unusual hibernoma (pale cell variant) in the subepidermal area of the nipple of a six-year-old bitch. Furthermore, an extensive literature review of hibernomas in animals was made. Physical examination revealed a nodular lesion in the subepidermal area of the third nipple of the left mammary chain. The histopathological findings included lobules of round to oval cells with abundant pale to eosinophilic cytoplasm, containing one or multiple optically empty vacuoles, consistent with nipple hibernoma. Immunohistochemically, the neoplastic cells were negative for cytokeratin AE1/AE3 and p53 but showed strong immunoreaction for vimentin and uncoupling protein-1, thus confirming the brown adipose tissue origin. Local recurrence was not detected after 18 months of follow-up. Hibernomas are rare and benign neoplastic lesions, originating from brown adipose tissue. Due to their histological and molecular resemblance with liposarcoma, a correct diagnosis of these neoplasms is required. In addition, the literature review suggests that hibernomas may present different features, according to species.

## Introduction

Hibernomas are rare soft tissue benign neoplasms that originate in brown fat (brown adipose tissue, BAT) (1). These tumors may originate from BAT remnants, ectopic growth or migration of brown adipose cells or aberrant differentiation of mesenchymal cells (2). BAT is a specialized form of adipose tissue specifically designed to generate heat in response to cold (nonshivering thermogenesis) and food ingestion (diet-induced thermogenesis). BAT heat production is activated whenever the organism requires extra heat, like in the postnatal period, during hibernation or when entering into a febrile state (3). The brown aspect of BAT is attributed to the large numbers of cellular mitochondria and abundant blood capillaries (4). In the human fetus, BAT has been identified in the interscapular area, posterior abdominal wall, suprailiac, and peripancreatic adipose tissue, and near autonomic ganglia. Thus, it is not surprising that hibernomas have often been reported in these locations. However, only a few hibernomas were reported in sites where brown fat is usually absent, such as breast or thigh, so far (5). The distribution of brown fat is not as well-characterized in dogs (6).

Currently, there are several reported cases of hibernomas in veterinary medicine, mostly in the orbital region of dogs (7). In the present study, we describe the clinical, pathological, and immunohistochemical features of a hibernoma located in the subepidermal area of the nipple in a bitch. Furthermore, an extensive literature review of hibernomas in animals was conducted.

## Case Description

A six-year-old intact female mixed breed dog was presented with acute pruritus of ears, muzzle, and abdomen. The dog has been previously vaccinated and treated for both internal and external parasites. The physical examination revealed a decreased diameter of the auditory canal of the left ear with inflammation and brown granular content with a strong rancid odor, compatible with the overgrowth of *Malassezia* spp. that was further confirmed by cytology. Erythema and papular skin lesions were present on the abdomen. An additional nodular lesion was detected in the subepidermal area of the third nipple of the left mammary chain. The owner was aware of this lesion for at least 1 year before the dog was presented to the hospital, without causing any pain or discomfort to the animal. The dog was treated with corticosteroids (prednisolone administered orally at 0.5 mg/kg BID for 3 days, followed by the same dosage SID for 3 days, and then QID for other 3 days) along with cleansing and topical application of Canaural® ear drops for the treatment of otitis externa. Excision of the mammary mass was advised and performed ~2 months later, after complete recovery of the dermatological signs.

The lesion was surgically removed, fixed in 10% neutral buffered formalin and submitted for histological examination.

For histology, the samples were cut and processed routinely, and sections were stained with hematoxylin and eosin (HE) and periodic acid-Schiff (PAS). Immunohistochemistry was performed using the Novolink™ Max-Polymer detection system (Novocastra, Newcastle, UK), employing the following monoclonal antisera: pan-cytokeratin (clone AE1/AE3, Zymed) diluted 1:50; vimentin (clone V9, Dako) diluted 1:100; uncoupling protein-1 (UCP1) (clone ab23841, Abcam) diluted 1:20 and p53 (clone BP53.12, Zymed) diluted 1:400. Positive controls consisted of sections of mouse perirenal adipose tissue (Supplementary Figure 1) and canine sebaceous gland adenocarcinoma (Supplementary Figure 2) known to express UCP1 and p53, respectively. A section of subcutaneous adipose tissue of a dog was used as negative control for UCP1 (Supplementary Figure 3).

Grossly, the tissue fragment was represented by a nipple, with an exophytic nodular and dense mass, measuring 12 mm × 6 mm × 4 mm. In cross-section, the lesion was well-delimited, partially encapsulated, with a homogenous appearance and white-yellow color.

**Figure 1:**
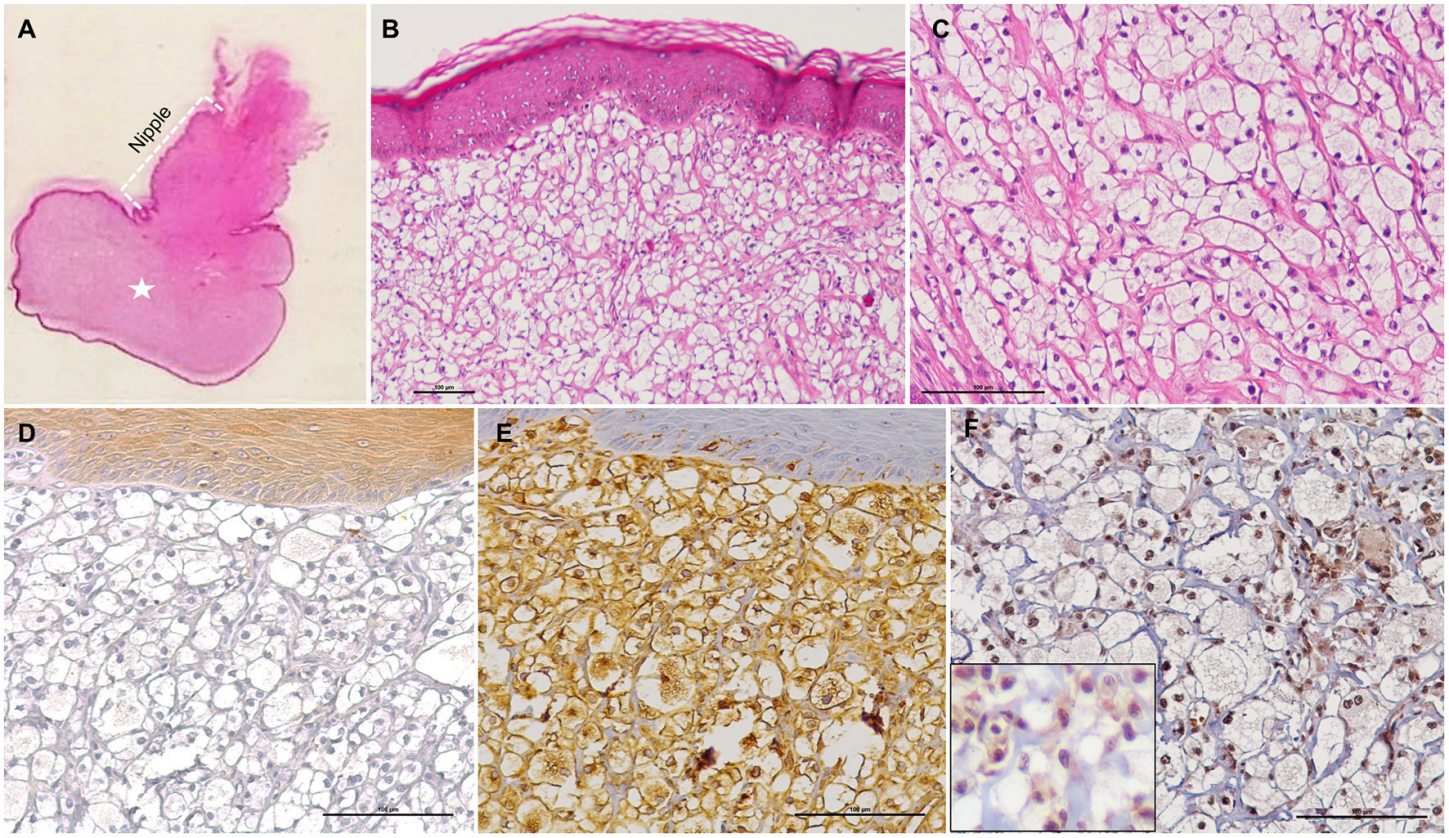
Histological and immunohistochemical features of a hibernoma (pale cell variant) in the subepidermal area of the nipple in a bitch. **(A)** The subepidermal area of the nipple is distended by an expansile, benign tumor (white asterisk), HE stain. **(B,C)** The tumor is composed of round to polygonal neoplastic brown fat cells arranged into sheets and poorly defined lobules, separated by a fine fibrovascular stroma. The neoplastic cells show abundant vacuolated cytoplasm, and mild anisocytosis and anisokaryosis, HE stain. **(D)** The neoplastic cells are negative for cytokeratin AE1/AE3, but positive for Vimentin **(E)** and UCP1 [**(F)** and higher magnification in the inset]. IHC.

Histological examination revealed a well-defined, partially encapsulated, multinodular and highly cellular neoplastic mass, effacing and expanding the subepidermal area of the nipple (Figure 1A). The tumor was composed of round to polygonal cells arranged in nests and delimited by thin fibrovascular stroma. The neoplastic cells presented variably distinct cellular borders, clear or pale eosinophilic cytoplasm, containing one or multiple optically empty vacuoles, mainly resembling lipocytes (Figure 1B). The vacuolar content was PAS-negative and was consistent with, although not proven to be, lipid. The nuclear:cytoplasm (N:C) ratio was low, and the nuclei were round to oval, central or occasionally eccentrically positioned, with fine granular chromatin and 1–2 small, basophilic nucleoli. Anisocytosis and anisokaryosis were mild, and the number of mitotic figures varied from 0 to 2 per high power field (0.237 mm^2^) (Figure 1C). Scattered mononuclear inflammatory cells, represented by small lymphocytes, plasma cells, and some macrophages were observed within the lesion. There were no evidence for vascular invasion of neoplastic cells.

The neoplastic cell population did not exhibit any immunoreactivity for cytokeratin AE1/AE3 (Figure 1D) and p53 (Supplementary Figure 4) but showed strong positive immunostaining for vimentin (Figure 1E) and UCP1 (Figure 1F).

Based on morphological, histochemical, and immunohistochemical findings, a diagnosis of a subepidermal nipple hibernoma was made.

## Outcome

No local recurrence was detected after 18 months of follow-up.

## Discussion

A hibernoma is a rare and benign soft tissue neoplasm of the brown adipose tissue (BAT). It was originally described and named “pseudolipoma” by Merkel, in 1906. In 1914, Gery Louis (8) derived the name hibernoma from the tumor's histological similarity to brown fat in hibernating animals.

BAT is present in both human and animal fetuses, being gradually replaced with white adipose tissue (WAT) with aging. However, it may persist in variable degrees throughout adult life, namely in the cervical and interscapular areas, axilla, mediastinum and periaortic and perirenal zones (3). In WAT, fatty acids are metabolized to produce energy in the form of adenosine triphosphate (ATP). In BAT instead, these are metabolized in the form of heat, due to the presence of abundant mitochondria and thermogenin/UCP1 (3). UCP1 is a specialized protein located in the inner mitochondrial membrane which uncouples mitochondrial fatty acid oxidation from ATP synthesis and dissipates energy of substrate oxidation as heat (9). Thus, in both humans and animals, immunohistochemistry for UCP1 has been considered a useful marker to differentiate between BAT and WAT and for diagnosing hibernomas (7, 10, 11). Despite being a valuable marker, UCP1 is not specific and exclusive for hibernomas, since a recent investigation concluded that it was expressed in 23 out of 25 canine liposarcomas, even though neoplastic cells showed reduced immunoreactivity intensity when compared with the positive control (canine abdominal brown adipose tissue) (12). In the present study, and in accordance with the findings reported by Ravi et al. (7), at the subcellular level UCP1 was immunoexpressed in both cytoplasm and nuclei of the neoplastic cells. Cinti et al. (13) demonstrated that UCP1 mRNA expression in rodent brown adipocytes had a positivity pattern very similar to that of the protein IHC localization. Furthermore, immunopositive cells were identified close to negative ones, and some cells showed nuclear staining, with or without cytoplasmic immunoreaction.

In veterinary medicine there are <20 reports of hibernomas in few animal species and in different anatomical sites (Table 1). In dogs, there seems to be a slight predilection in males older than 8 years, while in rats, females are mainly affected (Table 1). In addition, the periorbital region seems to be the most frequent site in dogs, whereas in rats, hibernomas are most often encountered intrathoracically, into the mediastinum (Table 1). The latter location may give rise to new inferences about the histogenesis of these tumors, at least in these two species. In rodents, BAT depots are described in this precise location (3) further suggesting that these lesions may originate from BAT remnants. Contrariwise, to the best of our knowledge, to date there are no reports on the presence of BAT in the periorbital region of animals. Thus, the description of hibernomas in sites that are assumed to be devoid of BAT seems to reinforce the hypothesis that abnormal mesenchymal differentiation may be related with the histogenesis of this neoplastic lesion. Previously, Champigny et al. (25) showed that the adrenergic stimulation of WAT taken from five different locations of adult dogs resulted in reactivation of WAT UCP, which is a classical feature of BAT. These authors postulated that tumors may originate from “dormant” BAT deposits that histologically present WAT-compatible morphology but are in fact constituted by brown preadipocyte cells. Nevertheless, in order to prove this hypothesis, the complete identification and distribution of BAT in dogs needs to be fully characterized.

**Table 1:** Literature available data concerning spontaneously occurring hibernomas in animals.

**Species**	**Breed**	**Gender Age**	**Location and gross characteristics**	**Associated clinical signs**	**Diagnostic methods performed**	**Main histopathological findings/Final Diagnosis**	**Follow-up**	**References**
Dog	Labrador Retriever	M, 9 m	Omentum 20 mm × 10 mm × 10 mm	None (incidental finding during exploratory laparotomy)	Histopathology: HE Prussian blue positive	No mitotic figures **Benign**	Clinically normal 3 months after the laparotomy	(14)
Dog	Pekingese	ND, 11 y	Orbit Well-demarcated, soft lobulated mass of unknown size	Unknown	Histopathology: HE			
	Mixed	M, 9 y	Third eyelid base and ventral orbit Well-demarcated soft, pale, lobulated mass 23 mm × 15 mm × 19 mm	Exophthalmos	TEM: Multivacuolated cells that displaced the nuclei eccentrically, containing variably sized lipid droplets; their quantity was inversely proportional to the number of existing mitochondria.			
	Labrador Retriever	M, 8 y	Retrobulbar orbital space	Unknown				
	Labrador Retriever	F, 10.5 y	Third eyelid base and ventral orbit Soft, tan lobulated mass 10 mm × 10 mm × 05 mm	Dorsal displacement of the globe		**Benign**	NA	(7)
	Terrier cross	M, 13 y	Orbital space and bulbar conjunctiva Tan, soft, lobulated mass 20 mm × 15 mm × 10 mm	Unknown	IHC: UCP1 (+++) MyoD1 (–/+) Miogenin (–/+)			
	Cocker Spaniel	M, 10.6 y	Orbit Soft, white lobulated mass 20 mm × 18 mm × 5 mm	Exophthalmos				
	Tibetan Spaniel	F, ND	Supraconjunctival and retrobulbar Soft, tan, lobulated mass 8 mm × 4 mm × 7 mm	Unknown				
Dog	German Shepherd mixed breed	M, 10 y	Ventral subconjunctiva of the left orbit 24 mm × 20 mm × 11 mm	Conjunctival hyperemia	Histology: HE IHC: UCP1 (+++) MyoD1 (–) Miogenin (–)	Intermittently encapsulated mass. Moderate cellular pleomorphism and rare mitotic figures. Incomplete excision. **Benign**	No signs of recurrence 14 months after the excision.	(15)
Dog	German Shepherd	M, 12 y	Within the femoral muscles of the right hindlimb Encapsulated mass composed of light brown tissues	Leg deformation and enlargement	Histology: HE IHC: S100 (++) CD31 (–)	No mitotic figures nor cell atypia	NA	(16)
Dog	Chihuaha	F, 11 y	Ventral conjunctiva	Third eyelid protrusion	Cytology: FNA Histology HE IHC: Cytokeratin (–) Vimentin (+) UCP1 (+)	Mild to moderate anisocytosis and anisokariosis MI = 2 × 10 HPF Rare macrophages containing brown and granular pigment.	No signs of recurrence 1 year after excision.	(6)
	Mixed breed	F, 9 y	Eyelid and retrobulbar space of the left eye	Exophtalmus		Mild to moderate anisocytosis and anisokariosis MI ≤ 1 × 10 HPF	NA	
Cat	Siamese	F, 13 y	Abdominal wall with adhesion to mesenteric margin of descendent colon. Soft, greasy and lobulated mass, gray to light brown in color. 10 mm × 10 mm	Lethargy and dehydration. Difficulty in defecation, constipation and abdominal guarding on palpation.	Histology: HE IHC: S-100 (++) (+++) Osteopontin: (+)	**Benign**	No signs of recurrence since the post-operative period.	(17)
Rat	ND	ND	NA	NA	NA	NA	NA	(18)
		F, 14–20 m (*n* = 3)	Intrathoracic, attached to the ventral surface of the spinal column and encircling the descending aorta.	Flaccid paralysis of hindlimbs	Histology: HE Oil red O positive	Frequent mitoses and presence of necrotic foci. Presence of macrophages laden with hemosiderin and lipochrome.		
Rat	Sprague Dawley	F, 26 m (*n* = 1)	Slightly soft, multilobulated, gray to red brown mass. From 37.5 cm × 20.0 cm × 20 cm to 50 mm × 35 mm × 30 mm.	None		In one animal, neoplastic cells invaded the vertebral marrow and separated the bone from dura mater. In another case, neoplastic cells invaded the capsular blood vessels. **Malignant**	NA	(19)
Rat	Wistar	M, 92 w	Intrathoracic mass, adherent to the musculature of the vertebral column	Piloerection Hypothermia Labored and gasping respiration	Histology: HE Fontana and Schmorl's stains	Infrequent mitoses Groups of pigment-laden macrophages (possibly lipofuscin, since positive for Fontana and Schmorl's stains)	The three animals were euthanized	(20)
	Wistar	M, ND	Interscapular subcutaneous mass 45 cm × 40 cm × 18 cm	Hypothermia Epistaxis Weight loss	TEM: Cells cytoplasm with numerous mithocondria, ample and vesicular RER and small to large neutral fat droplets.	Poorly defined mass		
	CD	F, ND	Intrathoracic mass Soft, pale brown mass 30 cm × 24 cm × 24 cm	Swollen eye, ocular and nasal discharges. Piloerection Labored and gasping respiration Paleness Hypothermia		Capsule invasion		
Rat	Fischer 344	NA	Subcutis	NA	NA	NA	NA	(21)
Rat	Sprague–Dawley	10 M and 4 F	Intrathoracic, circumscribing aorta and/or compressing lungs and mediastinal structures.		Necropsy Histology: HE	Twelve tumors were considered benign; two displayed malignant features.	Twelve out of the total animals were found dead or euthanized.	(22)
		15 M and 10 F	Thoracic cavity (13 M and 10 F) Abdominal cavity (2 M) Subcutaneous mammary gland fat (1 M)			Sixteen tumors were considered benign and nine malignant. Metastases to the lungs (intravascular emboli) and occasionally in the adrenal gland	Twelve were found dead or euthanized.	
		13 M and 10 F	Thoracic cavity (23), mammary gland region (4), or abdominal cavity (1).			Twenty-two tumors were considered malignant. Six animals presented metastatic lesions	Seventeen were found dead or euthanized.	
Rat	Sprague–Dawley Crl:CD(SD)IGS	M, 53 w	Intrathoracic, partially adherent to the aorta, esophagus and lung 25 mm × 30 mm × 35 mm	Labored respiration Decrease in food consumption Body weight loss	Histology: HE (Clusters of macrophages with lipofuscin and hemosiderin pigments were positive for PAS, Schmorl and Berlin blue stains) Oil red O positive IHC: UCP1 (++)	Multifocally, less differentiated areas with atypical cells (anisocitosis, marked pleomorphism, karyomegaly and multinucleation), frequent mitoses and necrotic foci were present. Capsule infiltration and vascular invasion were evident. Diffuse clusters of intralesional macrophages with lipofuscin and hemosiderin pigment. **Malignant**	Found dead	(11)
Goose		M, 2 y	Ventrolateral subconjuntiva of the right eye Large, raised, granular, pink-yellowish mass	None	Cytology: FNA Histology: HE Oil Red O positive PAS slightly positive Ziehl-Neelsen and Cresyl violet stain were negative TEM: The cytoplasm of neoplastic cells contained numerous lipid droplets and mitochondrias.	**Benign**	No signs of recurrence 12 months after the excision.	(24)

*M, male; F, female; w, week-old; m, months-old; y, years-old; MI, mitotic index; HPF, high power field, FNA, Fine needle aspiration; HE, haematoxylin and eosin; TEM, Transmission electron microscopy; IHC, immunohistochemistry; +, weakly positive; ++, moderately positive; +++, strongly positive; NA, non-available; ND, not disclosed*.

Herein, the tumor was peculiar not only for the associated anatomical site (nipple), but also for its superficial and subepidermal location. An identical lesion was described in 9-year old boy (26).

In the majority of cases, hibernomas present as asymptomatic, painless, and slowly growing neoplasms, that are usually detected either as a palpable mass or as incidental imaging or necropsy findings, similarly to those occurring in humans (27). According to the literature, when present, the clinical signs are often related to the mass expansion and subsequent adjacent tissue compression, which may further dictate the clinical course (Table 1).

In human medicine, six histological variants of hibernoma are described according to WHO classification: granular or eosinophilic, pale cell, lipoma-like, myxoid, spindle cell, and hybrid (28). Herein, we described a neoplastic lesion that was classified as a pale cell hibernoma. However, details regarding the histological subtype of hibernomas in animals are usually not disclosed in the literature, impairing further comparisons.

Although complete surgical excision is advised to avoid recurrence, most authors claim that these neoplasms do not have the propensity for local recurrence or aggressive behavior. Once again, the existing bibliography seems to be consensual: all cases reported in dogs had benign features, regardless of the anatomical site or whether the surgical excision was complete; irregular and poorly defined boundaries, partial encapsulation, presence of cytological atypia and frequent mitosis, foci of necrosis and invasion of adjacent tissues and vessels are characteristics of malignancy mainly seen in rats. In specific cases, these features eventually led to potential paraneoplastic systemic manifestations, such as marked weight loss (29). Gadea et al. (30) also described the effect of a hibernoma resection on human body composition and metabolism. One year after the resection, the patient gained 15 kg of body weight associated with visceral fat gain, exposing the patient to high risks of metabolic disorder.

Differential diagnosis that should be considered: residual or normal brown fat, which usually does not form a distinct mass; xanthogranulomatous (lipogranulomatous) inflammation, which normally presents with abundant foamy macrophages admixed with mature adipocytes and inflammatory and multinucleated giant cells, both foreign body and Touton types; classical lipoma, although adipocytes are not multivacuolated cells; lipoblastoma, normally composed of immature cells that resemble fetal adipose tissue in different development stages; and liposarcoma, which usually presents with infiltrative behavior, high cellular atypia and high mitotic activity.

P53 overexpression was observed in two human lesions, leading to the hypothesis that functional inactivation of the protein product of this gene may be important in the development of these tumors (23). Furthermore, in hibernomas of transgenic mice, a simian virus 40 (SV40)-transforming gene linked to specific regulatory regions of adipocytes was identified, and the binding of SV40 large T antigen to tumor suppressor genes, including p53 was reported (23). Nevertheless, in the current case, no p53 protein expression was identified amongst the neoplastic cells. Interestingly, in a 2-year carcinogenicity study with tofacitinib (an oral Janus kinase inhibitor for the treatment of rheumatoid arthritis), an increased incidence of hibernoma was noted in female rats. In this research, it was hypothesized that Janus kinase/Signal Transducer and Activator of Transcription inhibition in BAT along with sympathetic stimulation might contribute to the genesis of hibernomas (31). Both investigations call into question the simplicity of these neoplasms and highlight the possible metabolic complexity of hibernomas.

Despite the great abundance and long-life maintenance of the brown adipose tissue in animals, its neoplastic transformation appears to be a rarer event than in humans given the scarcity of cases reported in veterinary medicine. Additionally, following analysis of the existing literature, it was found that these neoplasms may present different features or behavior, according to species. Thus, a correct diagnosis as well as proper monitorization and documentation of hibernomas is needed in order to get insights into their putative metabolic impact, and to avoid unreasonable, radical therapeutic approaches and overtreatments.

## Data Availability Statement

The raw data supporting the conclusions of this article will be made available by the authors, without undue reservation.

## Ethics Statement

Ethical review and approval was not required for the animal study because it was not the case.

## Author Contributions

IA and FF contributed to the sample collection and performed the pathological studies and interpretation of the results. IA and CT analyzed the data and wrote the paper. MT and FF participated in immunohistochemical interpretation. FG contributed to the design, supervised the study, and helped to draft the manuscript. All authors read and approved the final manuscript.

## Conflict of Interest

The authors declare that the research was conducted in the absence of any commercial or financial relationships that could be construed as a potential conflict of interest.
