# Supplementary figures and images for: Nipple Hibernoma in a Dog: A Case Report With Literature Review
Source: Front Vet Sci. 2021 May 12;8:627288. doi: 10.3389/fvets.2021.627288 (PMC8149592; doi:10.3389/fvets.2021.627288)

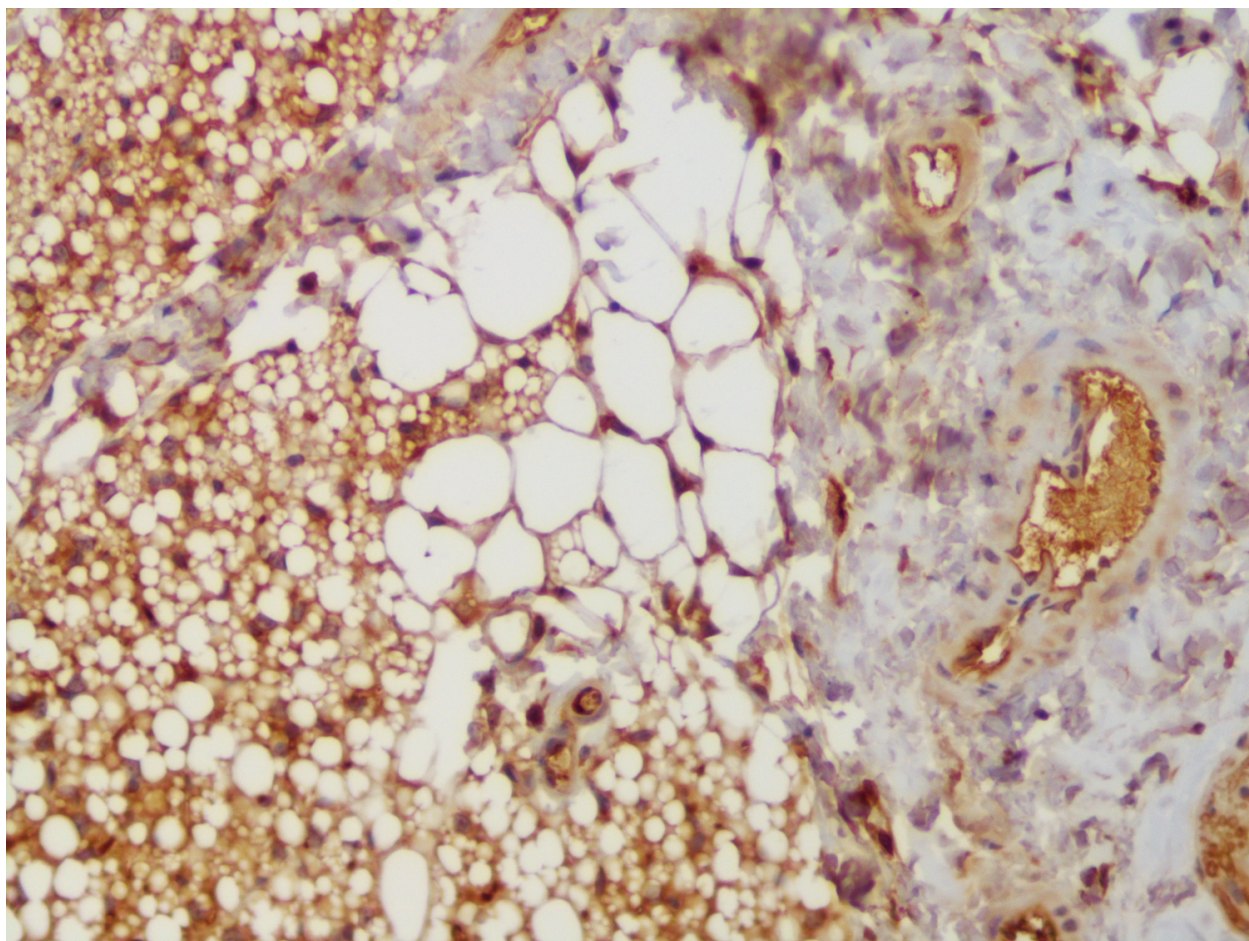

Supplementary Fig. 1. UCP1 positive immunorepression in mouse perirenal adipose tissue. IHC, x200.

Supplement: Supplementary file 1 [file Image_1.pdf]
