# Supplementary material for: Nipple Hibernoma in a Dog: A Case Report With Literature Review
Source: Front Vet Sci. 2021 May 12;8:627288. doi: 10.3389/fvets.2021.627288 (PMC8149592; doi:10.3389/fvets.2021.627288)
Supplement: Supplementary file 2 [file Image_2.pdf]

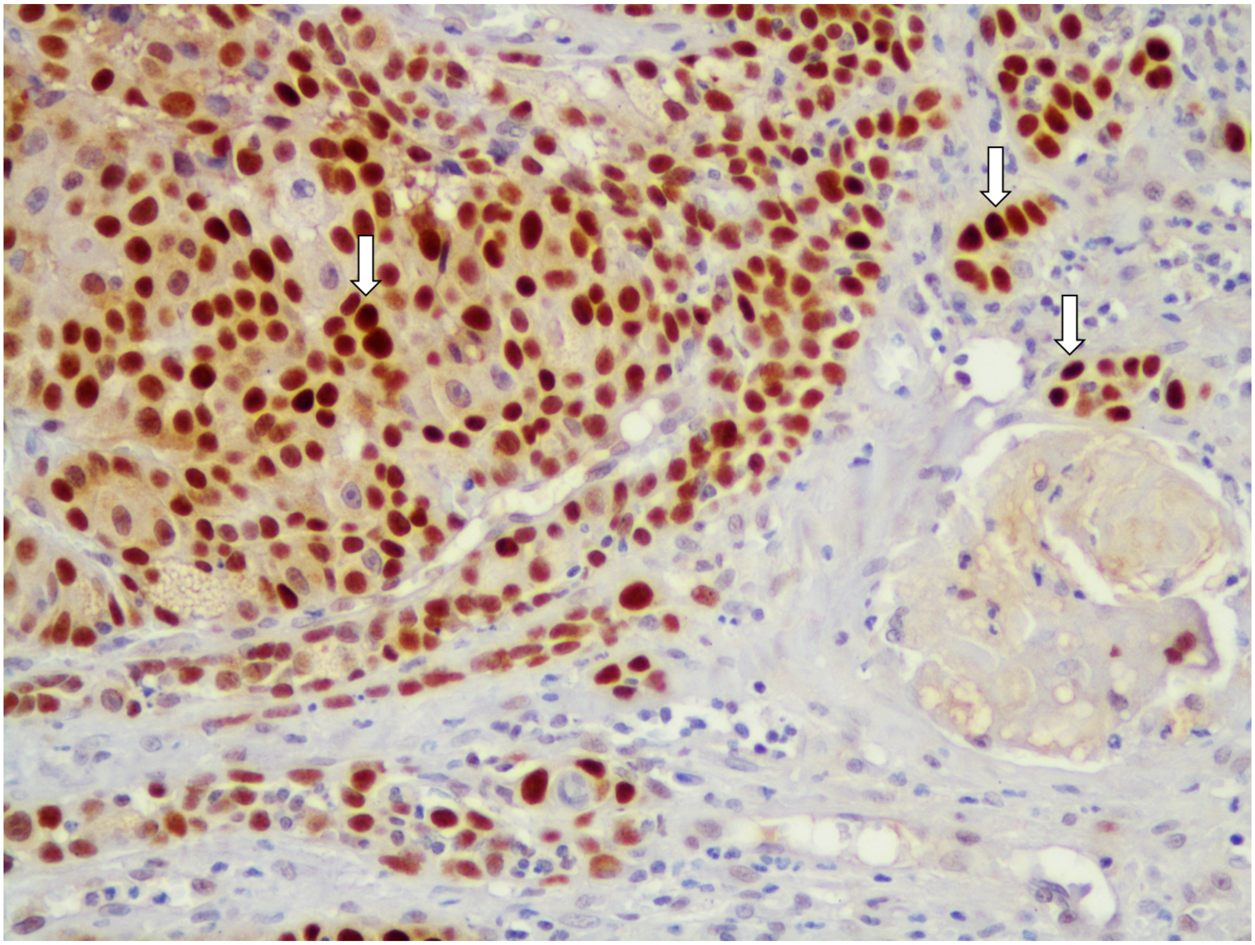

Supplementary Fig. 2. Canine sebaceous gland adenocarcinoma showing a strong p53 immunoreactivity of the nuclei (arrows). IHC, x200.
