# Supplementary material for: Nipple Hibernoma in a Dog: A Case Report With Literature Review
Source: Front Vet Sci. 2021 May 12;8:627288. doi: 10.3389/fvets.2021.627288 (PMC8149592; doi:10.3389/fvets.2021.627288)
Supplement: Supplementary file 3 [file Image_3.pdf]

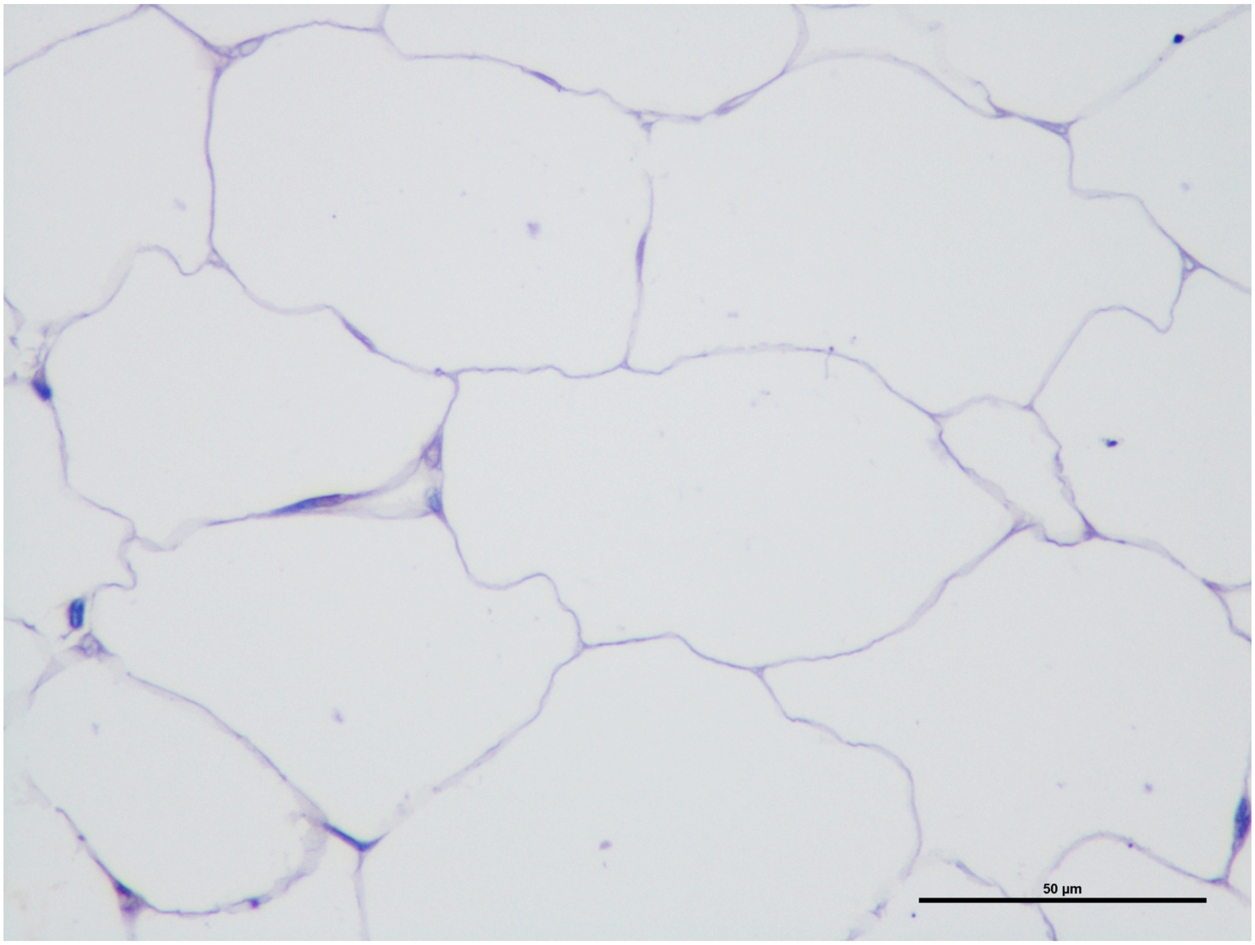

Supplementary Fig. 3. The subcutaneous adipose tissue in a dog. The adipocytes are negative for UCP1. IHC.
