# Supplementary material for: Nipple Hibernoma in a Dog: A Case Report With Literature Review
Source: Front Vet Sci. 2021 May 12;8:627288. doi: 10.3389/fvets.2021.627288 (PMC8149592; doi:10.3389/fvets.2021.627288)
Supplement: Supplementary file 4 [file Image_4.pdf]

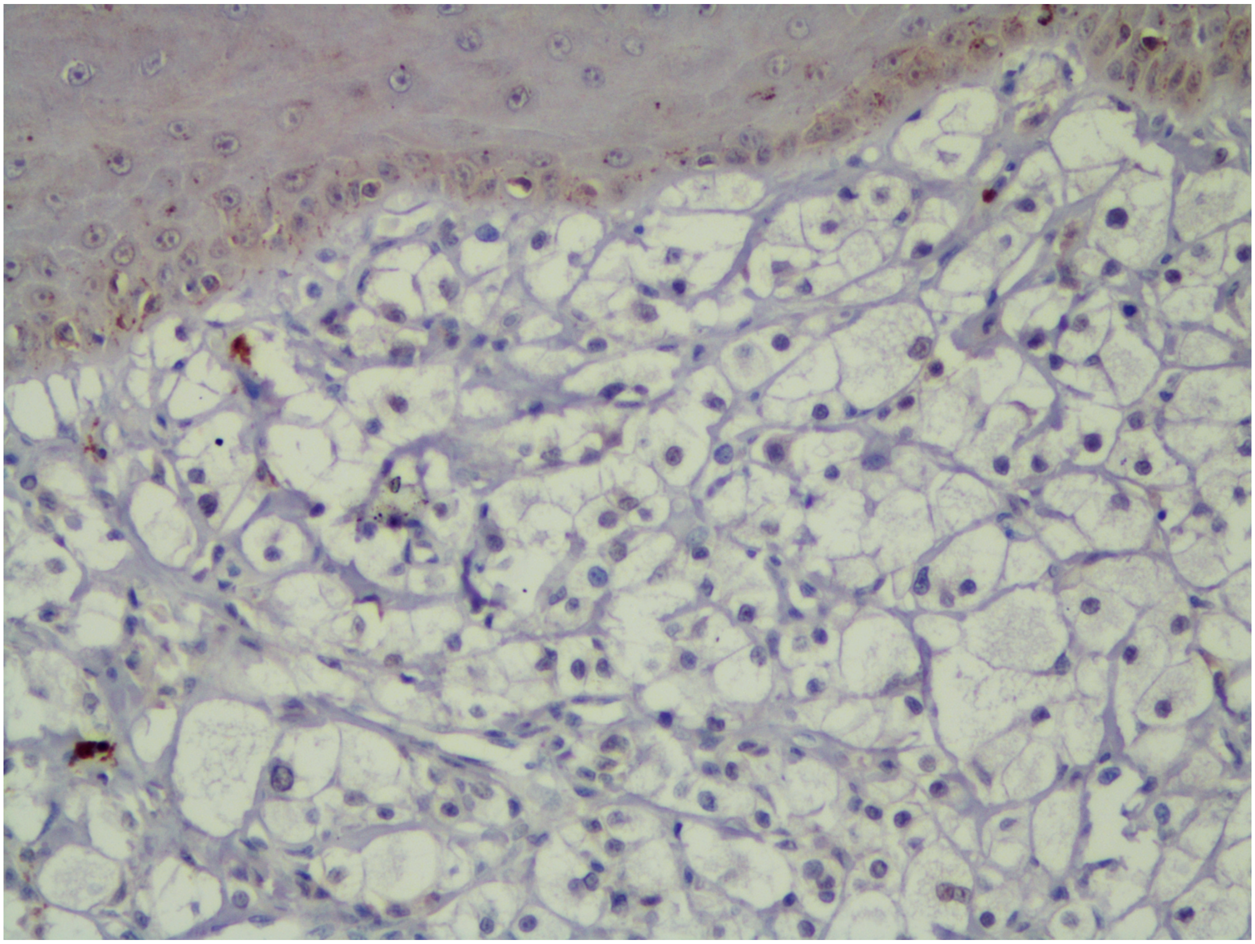

Supplementary Figure 4. The nuclei of hibernoma cells showing negative immunoreactivity for p53. IHC, x200.
